# Smoothing of the bivariate LOD score for non-normal quantitative traits

**DOI:** 10.1186/1471-2156-6-S1-S111

**Published:** 2005-12-30

**Authors:** Alfonso Buil, Thomas D Dyer, Laura Almasy, John Blangero

**Affiliations:** 1 Department of Genetics, Southwest Foundation for Biomedical Research, San Antonio, Texas, USA; 2 Institut de Recerca, Hospital de la Santa Creu i Sant Pau, Barcelona, Spain

## Abstract

Variance component analysis provides an efficient method for performing linkage analysis for quantitative traits. However, type I error of variance components-based likelihood ratio testing may be affected when phenotypic data are non-normally distributed (especially with high values of kurtosis). This results in inflated LOD scores when the normality assumption does not hold. Even though different solutions have been proposed to deal with this problem with univariate phenotypes, little work has been done in the multivariate case. We present an empirical approach to adjust the inflated LOD scores obtained from a bivariate phenotype that violates the assumption of normality.

Using the Collaborative Study on the Genetics of Alcoholism data available for the Genetic Analysis Workshop 14, we show how bivariate linkage analysis with leptokurtotic traits gives an inflated type I error. We perform a novel correction that achieves acceptable levels of type I error.

## Background

Variance component methods are very well suited to normally distributed phenotypes. However, when the phenotype under study is not normal, these methods tend to increase the type I error [1]. A robust LOD score correction has been developed to solve this problem for univariate phenotypes [2,3]. This approach takes advantage of a remarkable result: the distribution of the likelihood ratio statistic under model misspecification is equal to a constant times a  variate [4]. Therefore, a robust alternative to the likelihood ratio statistic is: Λ_*R *_= *k*Λ and the analogous robust LOD score is: LOD_R _= *k*LOD. That is, the robust LOD score is proportional to the LOD score under model misspecification. So the problem simplifies to the search for the constant of proportionality *k*.

Blangero et al. [3] proposes a method based on simulation to estimate this constant of proportionality. The idea is to generate a sample of the distribution of LOD scores for the non-normal trait under the null hypothesis and a sample of the asymptotic expected distribution of LOD scores for a normal trait. Because these two distributions should be proportional, an estimator of the constant of proportionality *k *can be obtained from a simple regression. To calculate the LOD scores for the non-normal trait, a set of independent random markers are simulated for every subject in the study. We call these LOD scores the observed LOD scores. On the other hand, the expected LOD scores are sampled from the asymptotic distribution of the test under normality.

The benefit of this approach over direct use of the empirical distribution of the LOD scores is that it results in a LOD statistic whose interpretation remains intact. Also, the calculation of *k *requires fewer replicates than the empirical LOD score distribution for small *p*-values.

Our aim is to find a similar smoothing correction for the bivariate phenotype. The Collaborative Study on the Genetics of Alcoholism (COGA) data contains two quantitative phenotypes that are clearly non-normally distributed: MXDRNK and CIGPKY. Any bivariate linkage analysis containing either of these phenotypes will provide biased LOD scores.

In this work we investigate four questions: First, can we find a robust LOD score approximately proportional to the LOD score under model misspecification with the bivariate phenotype? Second, will the proportionality be between the two degrees of freedom (2-df) LOD scores or between the equivalent one degree of freedom (1-df) LOD scores? Third, what is the lower boundary of the number of replicates required to achieve a good approximation of the slope? Lastly, which smoothing constant is most appropriate for use with the phenotypes of the COGA data?

## Methods

### Data

The COGA is a multicenter research project to detect and map susceptibility genes for alcohol dependence and related phenotypes [5]. We worked with the COGA sample data available for the Genetic Analysis Workshop 14 (GAW14), consisting of 143 extended families with 1,350 family members with clinical and demographic data. To avoid the problems of a mixed population of different ethnicities, we only used the subset of White non-Hispanic individuals (1,074 individuals in 119 families). This set of data contains 15 quantitative phenotypes. Two of them are behavioral measures of psychiatric interest: the "maximum number of drinks consumed in a 24 hours period" (MXDRNK) is related with alcoholism diagnosis and provides a quantitative measure to grade alcoholic and non-alcoholic individuals; the "number of packs of cigarettes per day for one year" (CIGPKY), is highly correlated with alcohol consumption. The other 13 quantitative phenotypes are electrophysiological traits that measure the neuroelectric activity generated in response to stimulus. Electrodes attached to the scalp of an individual with conductive gel record the event-related potentials (ERP). Various spatial and temporal characteristics differentiate the different ERPs. View Begleiter et al. [5] for a detailed description of these phenotypes. We used sex and age as covariates in all the bivariate models of this study.

### Transformation from a 2-df LOD score to a 1-df LOD score

Every LOD score has a direct match with a *p*-value. However, because the log likelihood ratio (LR) test where the LOD score comes from has a different distribution for univariate than for the bivariate phenotypes, these LOD scores will have different interpretations. The LR test in the univariate case follows a 1/2:1/2 mixture of a chi square distribution with 1 degree of freedom and a point mass at zero [6]. On the other hand, the null hypothesis in the bivariate case involves constraining to zero three parameters: the genetic correlation due to the quantitative trait locus (QTL) for both traits, and the genetic correlation at that QTL. Thus, the LR test for the bivariate case follows a 1/4:1/2:1/4 mixture of chi squares with 3 and 1 degrees of freedom and a point mass at zero, respectively [7]. We call the former a 1-degree of freedom LOD score (1-df LOD) and the later a 2-degrees of freedom LOD score (2-df LOD). To obtain the 1-df LOD equivalent to a given 2-df LOD, we follow two steps: first, we calculate the *p*-value corresponding to the 2-df LOD; and second, we calculate the 1-df LOD corresponding to that *p*-value.

### The LODADJ method

The SOLAR command "lodadj" implements the simulation method described in the "Background" section. We used this command with 27 bivariate phenotypes form the COGA data (all the possible pairs that include MXDRNK or CIGPKY) to generate repeated unlinked markers, calculate their respective LOD scores, and estimate the correction constant. We chose the pair of phenotypes MXDRNK and ttth3 (one of the electrophysiological measures) as the bivariate phenotype to perform the main simulation of linkage with unlinked markers, in which we ran 100,000 replicates. This experiment, with a huge amount of simulated markers, will serve to test the utility of the proposed method. At the same time, we calculated the slope constant for the 1-df LOD scores obtained from the transformation of the original 2-df LOD scores.

For the 26 remaining pairs of phenotypes, we calculated the smoothing slope from only 1,000 simulated markers, based on the results of the slope sampling experiment described below. Moreover, in these cases we only give the smoothing slope calculated from the 2-df LOD score.

### Slope sampling

Given the 100,000 observed LOD scores for the MXDRNK-ttth3 phenotype under the null model, we selected 100 random samples of varying size *n *(100, 250, 500, 750, 1,000, 2,000, 3,000, 4,000, 5,000 and 10,000) and calculated the regression slope between the observed and the expected LOD scores for each sample. Thus, we obtained a pool of 100 slopes for each value of *n*, and we calculated the mean and standard deviation of the slopes for each pool.

### Software

We used SOLAR [8,9] for simulation and linkage analyses. We then used the package R [10] to perform statistical analyses and to draw the plots.

## Results

The 13 electrophysiological measures are approximately normally distributed, with kurtosis ranging from 0.2 to 1. However, both MXDRNK and CIGPKY present clear non-normal distributions with high kurtosis: 6.3 and 8.4, respectively.

**Figure 1:**
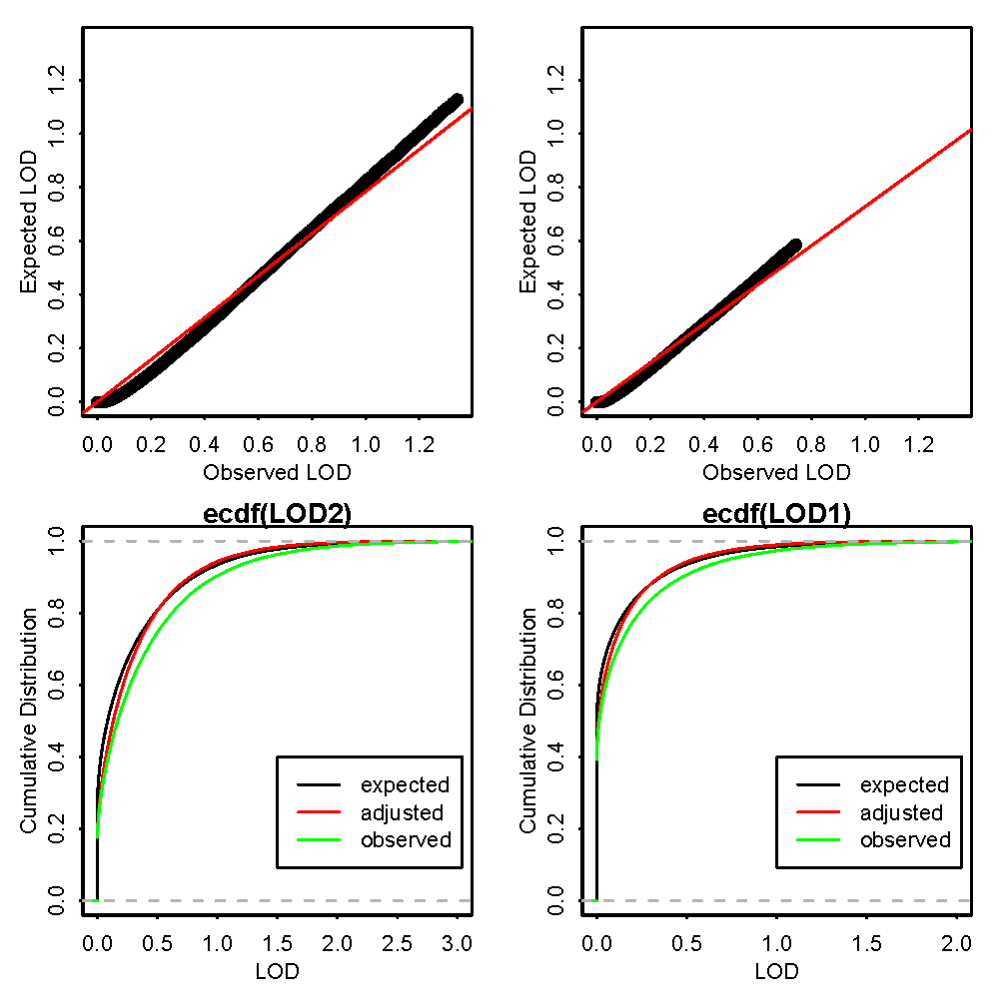
**Summary of the MXDRNK-ttth3 simulation. **Left: 2-df LOD scores. Right: 1-df LOD scores. Top: regression of the expected on the observed LOD scores. Bottom: cumulative distributions of the observed, expected and adjusted LOD scores.

Figure 1 shows a summary in four plots of the LOD adjustment results for the MXDRNK-ttth3 phenotype after the simulation of 100,000 unlinked markers. The plots in the first column are derived from the 2-df LOD scores; those in the second column are derived from the 1-df LOD scores. In the plots in the first row we show the regression of the observed LOD scores on the expected ones. In the plots in the second row we show the empirical cumulative distributions of the observed, expected and adjusted LOD scores.

The slopes are 0.78 and 0.73 for the 2-df LOD and 1-df LOD score, respectively. Thus, both regressions provide a similar fit. In both cases, we can detect a bias, the high values are over the mean, and the low values are under the mean. The plots of the cumulative distributions show also similar results for the 2-df and 1-df cases.

Table 1 summarizes the type I error rate for the observed and adjusted LOD scores for both the 1-df and the 2-df LOD score.

**Figure 2:**
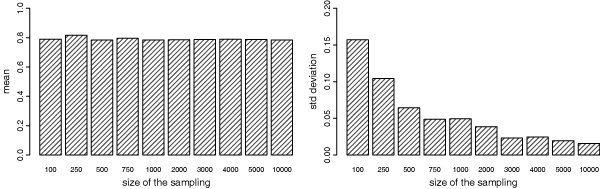
**Slope sampling results. **Mean (left) and standard deviation (right) of the correction slope for different values of *n*.

Figure 2 shows the means (on the left) and standard deviations (on the right) of the 100 slopes obtained for each value of *n*. The means stabilize quickly and the standard deviation decreases as the sample size increases. The standard deviation appears to become asymptotic around a sample size of 3,000 replicates but it is quite small with 1,000 replicates.

We chose this value (*n *= 1,000) to calculate the slope correction for all 27 bivariate phenotypes pairs of quantitative phenotypes of the COGA data containing MXDRNK or CYGPKY. Table 2 shows the smoothing correction constant for the different phenotype pairs and the corresponding 1-df LOD score equivalent to a LOD score of 3 in the case of normality.

## Discussion

Variance components linkage analysis is a powerful and flexible approach for the analysis of the genetic components of quantitative traits. Its main drawback is the increase of type I error when the traits of interest are not normally distributed. As in the univariate case, a smoothing correction for the LOD score is necessary in the bivariate case. In this work we have presented an empirical approach showing that the 2-df observed LOD score is approximately proportional to a more robust 2-df LOD score. It is important to note that the observed proportionality holds also for the equivalent 1-df LOD score. This fact is not surprising because the 2-df LOD and the equivalent 1-df LOD are nearly proportional. Thus, the correction for deviations from normality could be performed before or after the transformation to the 1-df LOD score. The smoothing approach presented here appears to work quite well, although it may be too conservative, as shown in Table 1.

Bivariate linkage analysis is computationally intensive and a simulation with 100,000 replicates is sometimes unfeasible. Our slope sampling experiment, however, indicates that 1,000 to 3,000 replicates should be enough to achieve a good estimation of the slope.

We calculated the correction slope for the MXDRNK and the CIGPKY phenotypes combined with the 13 electrophysiological measures of the COGA data. The range of the smoothing constants varies between 0.69 and 0.94.

If we consider a significant 1-df LOD score as being at least 3 (2-df LOD = 3.99), a slope correction of 0.8 requires a 2-df LOD = 4.95, equivalent 1-df LOD = 3.87 to be significant. Table 2 shows the 1-df LOD score required for each pair of phenotypes to achieve a significance equivalent to a 1-df LOD of 3 with two normally distributed phenotypes. This finding must be considered when using bivariate linkage analysis with this data.

## Conclusion

Variance component models for bivariate linkage analysis with at least one non-normally distributed phenotype give inflated type I error and then inflated LOD scores. A smoothing correction similar to the one available for univariate linkage analysis could be used to achieve a more accurate type I error and, thus, more reliable LOD scores.

The smoothing correction gives similar results when performed on the 2-df LOD scores or on the equivalent transformed 1-df LOD scores.

Compared with the direct use of the empirical distribution of the LOD scores, the calculation of the correction slope *k *proposed here requires fewer replicates for small *p*-values. Between 1,000 and 3,000 replicates are enough to obtain a reliable correction.

More studies are needed to evaluate the impact of this correction on the power to find linkage with a non-normally distributed bivariate phenotype.

## Abbreviations

CIGPKY: Number of packs of cigarettes per day for one year

COGA: Collaborative Study on the Genetics of Alcoholism

ERP: Event-related potential

GAW14: Genetic Analysis Workshop 14

LR: Likelihood ratio

MXDRNK: Maximum number of drinks consumed in a 24 hours period

QTL: Quantitative trait locus

## Authors' contributions

AB carried out the simulations and statistical analyses and drafted the manuscript. TDD participated in the design and implementations of the simulations. LA participated in the design of the study and performed a critical revision of the manuscript. JB conceived the study and participated in its design and coordination. All authors read and approved the final manuscript.

## Figures and Tables

**Table 1:** Summary of the type I error for the observed and adjusted LOD score

	2 degrees of freedom			1 degree of freedom		
		
Nominal α	LOD	observed	adjusted	LOD	observed	adjusted
0.1000	0.8088	0.1387	0.0914	0.3566	0.1387	0.0965
0.0500	1.1359	0.0744	0.0413	0.5875	0.0744	0.0429
0.0250	1.4613	0.0403	0.0184	0.8341	0.0403	0.0188
0.0100	1.8883	0.0174	0.0061	1.1752	0.0174	0.0060
0.0010	2.9481	0.0019	0.0003	2.0737	0.0019	0.0002
0.0001	3.9950	0.0002	0.0000	3.0034	0.0002	0.0000

**Table 2:** Correction slopes and significant 1-df LOD for 27 bivariate phenotypes of the COGA data

	Slope		1-df LOD significance	
		
Phenotype	MXDRNK	CIGPKY	MXDRNK	CIGPKY
CIGPKY	0.69		4.65	
ecb21	0.87	0.78	3.56	4.04
ntth1	0.8	0.76	3.89	4.15
ntth2	0.96	0.8	3.14	3.92
ntth3	0.89	0.82	3.41	3.77
ntth4	0.93	0.75	3.24	4.19
ttdt1	0.74	0.8	4.3	3.88
ttdt2	0.85	0.85	3.63	3.66
ttdt3	0.9	0.76	3.4	4.17
ttdt4	0.94	0.77	3.23	4.11
ttth1	0.75	0.71	4.2	4.46
ttth2	0.83	0.81	3.72	3.84
ttth3	0.84	0.82	3.68	3.8
ttth4	0.8	0.82	3.88	3.81
